# Current Insights Into Oligodendrocyte Metabolism and Its Power to Sculpt the Myelin Landscape

**DOI:** 10.3389/fncel.2022.892968

**Published:** 2022-04-28

**Authors:** Mohanlall Narine, Holly Colognato

**Affiliations:** ^1^Department of Pharmacological Sciences, Stony Brook University, Stony Brook, NY, United States; ^2^Department of Neurobiology, & Behavior, Stony Brook University, Stony Brook, NY, United States

**Keywords:** oligodendrocyte, myelin, metabolism, remyelination, glycolysis, lactate, oligodendrocyte progenitor cell (OPC)

## Abstract

Once believed to be part of the *nervenkitt* or “nerve glue” network in the central nervous system (CNS), oligodendroglial cells now have established roles in key neurological functions such as myelination, neuroprotection, and motor learning. More recently, oligodendroglia has become the subject of intense investigations aimed at understanding the contributions of its energetics to CNS physiology and pathology. In this review, we discuss the current understanding of oligodendroglial metabolism in regulating key stages of oligodendroglial development and health, its role in providing energy to neighboring cells such as neurons, as well as how alterations in oligodendroglial bioenergetics contribute to disease states. Importantly, we highlight how certain inputs can regulate oligodendroglial metabolism, including extrinsic and intrinsic mediators of cellular signaling, pharmacological compounds, and even dietary interventions. Lastly, we discuss emerging studies aimed at discovering the therapeutic potential of targeting components within oligodendroglial bioenergetic pathways.

## Introduction

The mammalian central nervous system (CNS) is a well-integrated network of cells comprising mostly neurons and glia (astrocytes, microglia, and oligodendrocytes). Neurons are responsible for the bulk of information processing in the CNS as well as complex behaviors and coordinated movements. Glia, on the other hand, designated “nervenkitt” or glue by Virchow and others, was first believed to be cells akin to connective tissue in the CNS with the function of providing structural support (Virchow, 1856). However, the last few decades have evolved our understanding of these specialized cells and has led to a “glial revolution”. For example, astrocytes are now known to facilitate neurotransmitter production, modulate synaptic plasticity, and, most recently, mediate neuroinflammation; whereas microglia, the resident immune cells of the CNS, monitor neuronal activity *via* their neurotransmitter surface receptors and in turn alter dendritic spine density to remodel synaptic circuitries (Paolicelli et al., 2011; Colonna and Butovsky, 2017; Weinhard et al., 2018). Oligodendrocytes (OLs), first discovered by Pio del Río Hortega who correctly purported them to be the Schwann cell equivalent in the brain and spinal cord, are the myelin-producing cells of the CNS (del Río-Hortega, 1921; Boullerne, 2016). Now, more than a century after the term “myelin” was coined by Virchow (Boullerne, 2016), OLs have been implicated in a wide array of white matter dysfunction, including those that have been recognized as contributing to behavioral disorders such as schizophrenia and bipolar disorder (Edgar and Sibille, 2012; Bellani et al., 2016). Recent work has also shown that OLs are involved in learning and memory, remodeling of neuronal circuits, and supporting axonal function and health, all of which rely, at least in part, on OL metabolic functions (Zatorre et al., 2012; Saab et al., 2013; Gibson et al., 2014; Moore et al., 2020). Given the importance of OL metabolism to OL function, in this review, we will describe what is known about the unique energetic demands posited by oligodendroglia, as well as explain how various metabolites can regulate oligodendroglia development and myelination. We then introduce the important role metabolism plays in removing toxic byproducts and how this “waste disposal” is essential for OL health and myelin maintenance. Finally, we conclude by examining the inputs that can regulate these bioenergetic properties, including extrinsic and intrinsic mediators of cellular signaling, pharmacological compounds, and even diet. Together we suggest that the emerging knowledge of OL bioenergetics will provide new ways to develop therapeutics that are beneficial to OL, and hence overall CNS function.

## Background

Oligodendrogenesis begins as early as E12.5 during the embryonic development of the rodent CNS (Warf et al., 1991). Within the ventricular zone of the neural tube, neuroepithelial cells are transformed into radial glial cells that further differentiate into intermediate progenitor cells, then oligodendrocyte progenitor cells (OPCs; Rowitch and Kriegstein, 2010). Specifically timed waves of OPCs begin to populate the CNS in a caudal to rostral manner, from the spinal cord to the forebrain, using their processes to sample and occupy vacant spaces (Kirby et al., 2006). Upon receiving differentiation-promoting cues, which remain to be fully elucidated, a subset of OPCs then differentiate into OLs that engage in wrapping axons with their lipid-rich myelin membrane (the process known as myelination). Myelin serves both to provide metabolic support and to promote the establishment of saltatory conduction, thus ensuring both neuronal health as well as fast and efficient impulse propagation along axons (Simons and Nave, 2015).

Some of the well-known factors governing OL development stem from transcriptional regulation as well as extracellular signaling mechanisms. For instance, transcription factors *Id2* and *Id4* have been shown to regulate OPC proliferation and differentiation (Kondo and Raff, 2000; Wang et al., 2001). Other transcription factors such as myelin gene regulatory factor (*Myrf*) are essential for CNS myelination such that when the membrane-bound Myrf protein undergoes an activating cleavage event, its cleavage product can localize into the nucleus to bind and enhance the expression of pro-myelin genes such as *Plp1, Mbp, Mag, and Trf* (Bujalka et al., 2013). The extracellular matrix protein laminin, on the other hand, has been shown to regulate OPC survival and the timing of myelination *via* activation of the Fyn kinase pathway as well as promote OPC migration and survival *via* focal adhesion kinase (FAK) mediated signaling (Relucio et al., 2009, 2012; Suzuki et al., 2019). All these events, however, require appropriate resources to support the biosynthetic pathways that are needed for progression through the cell cycle, differentiation, myelination, and interactions with cellular partners. Oligodendroglial metabolism is therefore critical in governing all aspects of key cellular dynamics, with elements such as energy production, lipid metabolites, and the production of reactive oxygen species closely impacting oligodendroglial development.

## Oligodendrocyte Metabolism

Cellular metabolism involves the sum of all biochemical processes involved in producing or consuming energy. Metabolic pathways largely fall into three distinct categories: anabolic, catabolic, or waste disposal. Anabolic pathways are a series of enzyme-catalyzed reactions that require energy inputs and carbon backbones to synthesize complex macromolecules such as proteins, carbohydrates, and lipids, whereas catabolic reactions ultimately produce energy by sequential degradation of large molecules into their smaller constituents: proteins into amino acids, carbohydrates into sugars, and lipids into fatty acids. Waste disposal mechanisms remove toxic byproducts and, together with total cellular metabolism, help to coordinate the optimal energetic balance needed for all cellular activities (Deberardinis and Thompson, 2012).

OLs generate the myelin sheaths that comprise a large fraction of the white matter, which itself is approximately half the total volume of the human brain (Herndon et al., 1998). One OL can myelinate up to 50 axons, with as many as 150 layers of compacted myelin membrane per segment (Baumann and Pham-Dinh, 2001; Fields, 2010). As could be imagined by this large cellular expansion, the energetic demands of OLs are substantial. In white matter, an estimated 82.3% of energy demands are associated with non-signaling activities by glia cells such as protein and lipid synthesis, oligonucleotide turnover, and cytoskeleton remodeling, compared to signaling activities (i.e., action potential firing; Yu et al., 2017; Dienel, 2018). Furthermore, a theoretical minimum of 6.84 × 10^21^ ATP molecules (the energy currency of the cell) are needed to produce 1 g of total myelin *protein* and 48-fold that per gram *lipid* of the myelin membrane. The benefit of this extensive upfront ATP expenditure is reduced energy usage during action potential propagation, with a predicted 23% savings in overall ATP needed when compared to unmyelinated axons (Harris and Attwell, 2012). Given the critical role that energy production plays in proper OL function and overall brain energy demand, it is important to understand the underlying metabolic components at work and how they may be used to counter oligodendropathies and demyelinating diseases.

### Oxidative Glycolysis in OL

**Figure 1:**
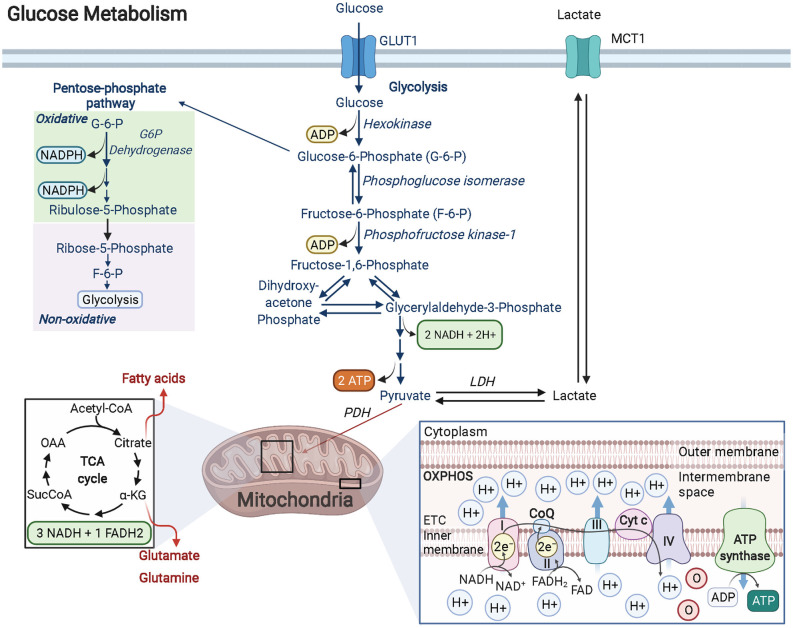
Glucose metabolism in OPCs/OLs. Glucose is imported into the cell by GLUT transporters (GLUT1 is the most abundant GLUT transporter in OLs). Glucose is transformed into G-6-P by the rate-limiting enzyme hexokinase. G-6-P can enter the pentose phosphate pathway (PPP) or continue down the glycolytic pathway. In OPCs and OLs, there is significant PPP activity when compared to other cell types in the brain. The PPP pathway produces NADPH which is essential for cholesterol synthesis (for lipids) and for enhancing antioxidant activity. Furthermore, PPP generates ribose-5-phosphate (R-5-P) which is the backbone used in nucleotide synthesis and is critical in maintaining the proliferative function of cells like OPCs. R-5-P can be converted to F-6-P and enter back into the glycolytic pathway to generate a net of 2 ATP and 2 NADH molecules as well as pyruvate. Pyruvate is then converted to AcCoA by the PDH complex and enters the TCA cycle. The metabolites formed from the TCA cycle become electron donors of the electron transport chain, driving the hydrogen potential difference across the inner mitochondrial membrane to power ATP synthase and produce ATP. Created with BioRender.com.

Glucose is the preferred fuel in the brain and is primarily catabolized *via* aerobic glycolysis (Dienel, 2018). During oxidative glycolysis, glucose first enters the cell and is irreversibly phosphorylated by hexokinase to glucose-6-phosphate (G-6-P). The G-6-P metabolite can then continue down the glycolytic pathway, shuttle into the pentose phosphate pathway (PPP), or be stored as glycogen (Figure 1). Both the glycolytic and PPP pathways are involved in the production of pyruvate, a critical metabolite that is converted to acetyl coenzyme A (AcCoA) by the pyruvate dehydrogenase (PDH) enzyme complex. AcCoA enters the citric acid cycle (TCA) in the mitochondria to generate the electron carrier metabolites NADH and FADH_2_ upon cycle completion. Both NADH and FADH_2_ enter the electron transport chain to produce an estimated net 30 ATP molecules (net amount is lower than theoretical estimates after accounting for proton leak) *via* oxidative phosphorylation (OXPHOS). Notably, the amount of ATP produced by OXPHOS per molecule of glucose is substantially greater than the net 2 ATP molecules generated solely through glycolysis (Dienel, 2018). Remarkably, OLs have been shown to prefer glycolysis over OXPHOS in spite of their high energetic demands. A recent study focused on characterizing the distinct bioenergetic properties of both *human-*derived adult OPCs and OLs, demonstrated that under favorable and nutrient-rich conditions, OPCs and OLs both rely significantly more on glycolysis than OXPHOS to generate ATP (Rone et al., 2016). Additionally, when subjected to stress conditions like those observed in the demyelinating disease, multiple sclerosis (MS), OLs begin retracting their processes to destabilize the compact axonal myelin sheath, which is thought to limit cell death by entering a less-energy intensive, low metabolic state (glycolysis). This response is perhaps favorable in the short-term, preserving existing OLs should they experience conditions conducive for a metabolic rebound. In contrast, OPCs are not capable of sufficiently reducing their metabolic rate as these cells require substantially more ATP. As a result, OPCs begin to rapidly die under stress. Surprisingly, developing *rodent* OPCs have been found to be *more* metabolically active compared to *rodent* OLs, capable of elevating both OXPHOS and glycolysis, with both rodent OPCs and OLs producing more ATP than their human counterparts (Rone et al., 2016).

A central role for glycolysis in OLs was firmly established by studying mice with an inducible knockout for the *Cox10* gene in immature OLs from birth. *Cox10* encodes for cytochrome *c* oxidase (COX—also known as complex IV), which is the terminal electron acceptor in the electron transport chain of the mitochondria that helps to establish the proton gradient needed for ATP generation *via* OXPHOS. Despite confirmed mitochondrial dysfunction, mutant mice (Cox10^flox/flox^*Cnp1^Cre/+^) displayed no signs of demyelination, oligodendroglial pathology, or white matter abnormalities, even at 9 months of age (Fünfschilling et al., 2012). Lactate levels, however, were found to be significantly increased. Lactate can be generated from the reaction between pyruvate and NADH, catalyzed by lactate dehydrogenase (LDH). Given that the majority of lactate comes from glycolytic pyruvate and that it is secreted from the cell (*via* monocarboxylate transporters, MCTs), lactate is often used as a readout for glycolytic activity (Rogatzki et al., 2015). In other words, OLs were demonstrated to survive and adequately function by using glycolysis to generate their needed energy, although it should be noted that the energy required for mature OL maintenance is lower than developing OLs. Nevertheless, even with lower overall ATP yields, the kinetics of glycolysis is much faster than complete glucose oxidation (glycolysis + OXPHOS; Dienel, 2018). Additionally, the increase in lactate production from enhanced glycolysis can benefit the underlying ensheathed axons since myelin wrapping enables the formation of “myelinic channels” for which lactate and other trophic factors can be shuttled to axons to maintain axonal health (Simons and Nave, 2015). Thus, the high energy needs of neurons during action potential signaling, calculated to be 4.1 × 10^5^ ATP/s for an axon diameter of 0.76 μm and 1.8 × 10^6^ ATP/s for a larger 1.26 μm axon diameter firing at physiological rates of 3 Hz and 8 Hz, respectively (Harris and Attwell, 2012), can be supported by this influx of lactate, which can be readily converted to pyruvate *via* LDH, followed by conversion to AcCoA by pyruvate dehydrogenase. AcCoA can then enter the TCA cycle to ultimately produce the byproducts NADH and FADH_2_ that will feed into complex 1 and complex 2 of the electron transport chain, respectively, fueling OXPHOS-linked ATP production, ultimately offering a faster way for neurons to produce ATP that bypasses the upstream glycolytic pathway altogether (Figure 1). The ability of lactate-derived ATP to meet energy demands in the brain is reminiscent of astrocytic glycogen-derived lactate that has been shown to help maintain ATP in an exhaustive-exercising brain model, thus protecting neurons from cell death (Matsui et al., 2017). Lactate’s role in OLs may go beyond supporting neurons as evidence suggests that OLs can also *import* this metabolite through MCT1 transporters, providing a way to support oligodendrocyte development and myelination when glucose concentrations are low (Rinholm et al., 2011). OLs also oxidize lactate at a greater rate than neurons and astrocytes do for lipid synthesis, which may contribute to the preference for glycolysis in these cells (Sánchez-Abarca et al., 2001).

However, it is important to note that other compounds regulate axonal metabolism through their actions within OLs. In particular, sirtuin 2 (SIRT2), a member of the sirtuin family of signaling proteins involved in metabolic regulation, has recently been shown to be present in OL exosomes that readily cross the periaxonal space and enter axons, where the release of SIRT2 (which is undetectable in neurons) acts on mitochondrial proteins to deacetylate them and increase axonal ATP levels, even in the presence of lactate and high glucose containing media (Chamberlain et al., 2021).

Disruption in OL glycolytic activity is also thought to contribute to many diseases, including Alzheimer’s disease (AD). Early AD is accompanied by white matter loss, OL death, and axonal degeneration in both human AD patients and AD animal models; these early events have all been linked to mitochondrial dysfunction. Further investigation into the role OL stress plays in AD neuropathology determined that dynamin-related protein-1 (Drp1) was activated, leading to mitochondrial fission and fragmentation, causing impaired bioenergetics and suppression of glycolytic activity in OLs *via* inhibition of the rate-limiting enzyme hexokinase-1. In turn, the NLRP3 inflammasome (a multimeric protein complex) was activated, which promotes proinflammatory programmed cell death. However, a genetic knockout of Drp1 restored glycolytic rates in mature OLs of AD mice, which prevented myelin loss, reduced axonal degeneration, and improved cognitive performance. Thus, this study provided strong evidence that correctly regulated OL glycolysis plays an important role in CNS disease (Zhang et al., 2020).

### Pentose Phosphate Pathway

The PPP is a biosynthetic pathway (non-ATP producing) in cells that branches from glycolysis after glucose is metabolized to G-6-P by the enzyme hexokinase. Once committed, G-6-P enters the oxidative, irreversible state of the PPP pathway where in a series of stepwise reactions, it forms 6-phosphogluconate, ribulose-5-phosphate (Ru-5-P), and 2 molecules of NADPH. In the non-oxidative, reversible arm of the PPP, Ru-5-P is converted to ribose-5-phosphate (R-5-P) to provide the carbon backbone needed for nucleotide synthesis (Patra and Hay, 2014). Additionally, other PPP metabolites such as fructose-6-phosphate (F-6-P) and glyceraldehyde-3-phosphate (G-3-P) can shuttle back into the glycolytic pathway or, in the case of F-6-P, can be converted back into G-6-P and replenish oxidative PPP by generating more NADPH (Figure 1), which in turn can provide redox power for myelin lipid synthesis or bind with the enzyme catalase to enhance antioxidant activity within the cell (Kirkman et al., 1987; Berg et al., 2002; Kowalik et al., 2017).

The metabolic flux of specific metabolites can be analyzed within cells using 13-carbon (^13^C) labeling techniques. With this approach, Amaral et al. (2016) assessed [1,2-^13^C] glucose in differentiated OLs to better understand the metabolic pathways involved in glucose catabolism. They found significant glucose metabolism in OLs, with 10%–15% being metabolized *via* the PPP pathway, a rate similar to that seen in astrocytes. Additionally, they noted that the F-6-P and G-3-P derived from R-5-P of the PPP can enter the glycolytic pathway to produce pyruvate, which gets carboxylated to oxaloacetate (the last metabolite generated in the TCA that forms citrate after condensation with AcCoA) by the enzyme pyruvate carboxylase (PC; Berg et al., 2002; Amaral et al., 2016). The generation of oxaloacetate by pyruvate carboxylation allows alpha-ketoglutarate (α-KG, one of the intermediates in the TCA) to leave the cycle and enter the cytosol to form glutamate, or in the presence of glutamine synthetase (GS), make the amino acid glutamine. This finding has a profound impact on OL metabolic functions as this glutamate-glutamine cycle has previously only been described in astrocytes, where it aids neurons in synthesizing the most abundant excitatory and inhibitory neurotransmitters in the brain, glutamate, and GABA, respectively (Hertz, 2013). Although it is yet to be seen if glutamine release occurs in OLs, it is possible that myelinic channels formed during wrapping can shuttle glutamate/glutamine to neurons and assist in neuronal signaling and more specifically provide feedback for adaptive myelination. It is important to note that in addition to elevated PPP, Amaral et al. (2016) observed high OL mitochondrial activity (TCA activity) but the level of OXPHOS was not reported.

Similar to OLs, OPCs display high glucose catabolism in the PPP pathway. When labeling OPCs with ^14^Carbon glucose (^14^C-glucose) to assess the flux of glucose through different metabolic pathways in the postnatal rat brain, the PPP was reported to be two-fold higher than in type 1 astrocytes, 10-fold higher than in type 2 astrocytes, and at least four-fold higher than in neurons (Sánchez-Abarca et al., 2001). This is not surprising given that OPCs are highly proliferative cells, especially during development and in the subventricular zone of adult brains (Spitzer et al., 2019), and thus require ample nutrients for cell cycle progression. This requirement is most pronounced within the late G1/S phase of the cycle, where the non-oxidative branch of the PPP synthesizes R-5-P, the precursor for nucleotides adenine and guanine, to aid in DNA synthesis before mitosis and complete cell division can occur (Khedkar et al., 2016).

Inhibition of PPP in OLs with supra concentration of the G-6-P inhibitor dehydroepiandrosterone (DHEA) compromises NADPH supply and causes significant OL death and decreases in myelin. Similar results are also observed when treating both OPCs and OLs with the NADPH antimetabolite 6-aminonicotinamide (6AN). These findings were attributed to a disruption in NADPH-linked antioxidant activity and treatment with reduced glutathione (a naturally occurring antioxidant) was able to rescue the deficits observed, thereby, highlighting the importance of PPP activity and the many metabolites it produces in oligodendroglia (Kilanczyk et al., 2016).

## Reactive Oxygen Species

Reactive oxygen species (ROS) such as superoxide and hydrogen peroxide, are derivatives of molecular oxygen generated from by-products of aerobic metabolism. The majority of ROS (~90%) come from mitochondrial activity during OXPHOS where electrons can escape the inner mitochondrial membrane to generate free radicals, oxygen molecules with highly reactive unpaired electrons. However, ROS can also be generated by lipid metabolism within peroxisomes, through enzymatic activity within the cytosol, or by plasma membrane proteins such as NADPH oxidase (Lambert and Brand, 2009; del Río-Hortega, 2012; del Río and López-Huertas, 2016; Pizzino et al., 2017; Checa and Aran, 2020). Excess ROS can cause oxidative stress and lead to cellular damage whereas physiological levels have key roles as cell signaling molecules needed for normal biological processes (Pizzino et al., 2017; Sies and Jones, 2020). In the case of excess ROS, cells rely on several mechanisms to eliminate free radicals such as the antioxidant system and scavengers, which are stable molecules that donate electrons to free radicals in order to neutralize them (Lobo et al., 2010). The enzyme superoxide dismutase (SOD) and the tripeptide glutathione (GSH) are among the most abundant endogenous antioxidants of the cell. SOD works by catalyzing the conversion of superoxide to hydrogen peroxide, which further gets converted to water *via* the catalase enzyme, whose activity is enhanced by NADPH (a product of the PPP pathway—see Section “Pentose Phosphate Pathway”) binding (Slemmer et al., 2008; Fernandez-Marcos and Nóbrega-Pereira, 2016). NADPH is also used as a cofactor by glutathione reductase to generate reduced GSH that defends against oxidative stress (Slemmer et al., 2008; Gansemer et al., 2020). Interestingly, oligodendroglia express low levels of both manganese SOD (the major detoxifying SOD in the cell) and GSH, making them particularly vulnerable to damage from oxidative stress, a phenomenon that has been observed in multiple white matter pathologies such as spinal cord injury (SCI), MS, schizophrenia (SCZ), Parkinson’s disease (PD), and Alzheimer’s disease (AD; Thorburne and Juurlink, 1996; Pinteaux et al., 1998; Holley et al., 2011; Spaas et al., 2021). Mutations in the human *SOD1* gene are associated with familial amyotrophic lateral sclerosis (ALS), and result in substantially increased ROS concentrations in the nervous system (Said Ahmed et al., 2000). Recent work has revealed that in ALS, loss of SOD activity in OLs leads to demyelination, downregulation of MCT1 transporters (transporter that shuttles the critical metabolite lactate to neurons), and subsequent motor neuron degeneration (Kim et al., 2019). Conversely, overexpression of the human *SOD1* gene in transgenic mice reduced oxidative stress-mediated death in OPCs isolated from cerebral cortices. In the same study, OPC proliferation was found to be increased and differentiation was accelerated, with the resulting mature OLs being more resistant to cell death when compared to wild type (WT) controls (Veiga et al., 2011).

Similar to SOD dysfunction, deficits in GSH synthesis resulted in impaired OPC proliferation and maturation in a model of SCZ, where anomalies in white matter integrity have been reported in the patient population (Monin et al., 2015). Intriguingly, trinucleotide repeat polymorphisms in the *GCLC* gene, which encodes the catalytic subunit of glutamine cysteine ligase (GCLC), the rate-limiting enzyme of GSH synthesis, have been observed in SCZ patients (Gysin et al., 2007). Knockdown of *GCLC* in cultured OPCs showed a ~28% decrease in total GSH levels and a subsequent ~29 reduction in OPC proliferation compared to WT controls. Additionally, reduced GSH leads to a dramatic decrease of (~57.2%) in the number of MBP+, mature OLs. Further, an analysis of oligodendroglial dynamics in a mouse that lacks the modifier subunit of glutamine cysteine ligase (GCLM-KO), a mouse model that displays SCZ behavioral phenotypes, presented similar reductions in GSH and OPC proliferation and maturation levels at the peripubertal age (Monin et al., 2015). These and other studies have begun to uncover the key role that oligodendroglial metabolism may play in behavioral disorders.

A fine balance in redox reactions is critical for cell survival and function, as ROS at physiological levels can behave as signaling molecules that mitigate stress responses, DNA damage, and promote cellular differentiation (Sies, 2015; Checa and Aran, 2020). In fact, treatment of the human oligodendroglial cell line MO3.13 (which expresses phenotypic markers of OPCs) with hydrogen peroxide at low levels (200 μM) displayed significant *increases* in MBP RNA and protein levels. Moreover, hydrogen peroxide treatment induced the activation of the extracellular signal-regulated kinase 1–2 (ERK 1/2) and of cyclic AMP responsive element binding protein-1 (CREB-1), known regulators of OL differentiation (Sato-Bigbee et al., 1999; Fyffe-Maricich et al., 2011; Accetta et al., 2016). NADPH oxidase (NOX), a membrane bound enzyme that generates ROS, also increased MBP mRNA levels when treated with low concentrations of hydrogen peroxide, and silencing of NOX genes (*NOX3* and *NOX5*) in MO3.13 cells inhibited MBP and Olig2 mRNA expression, and OL differentiation. Together these studies have highlighted the important role of redox signaling in oligodendroglia (Accetta et al., 2016; Vilchis-Landeros et al., 2020).

## Myelin Lipid Metabolism

One result of myelin wrapping (i.e., myelination around axon segments) is the rapid transmission of action potentials along neurons *via* saltatory conduction. Most but not all neurons in white matter tracts of the nervous system eventually become myelinated, enabling fast and reliable information processing for proper circuitry function (Almeida and Lyons, 2017). In the CNS, there is continued myelin sheath remodeling and lipid turnover mediated by OLs, which produce ~40% of the total lipids synthesized in the human brain, most of which is used for forming myelin membrane (Schmitt et al., 2015). Interestingly, the molecular makeup of the *myelin* membrane itself differs drastically from other cellular membranes in that it contains ~70% of its dry weight as lipids compared to 25%–50% in other cells (Cooper, 2000; Jahn et al., 2009). While myelin is not unique to the CNS [Schwann cells wrap myelin around axons in the peripheral nervous system (PNS)], the *lipid* composition of myelin throughout the nervous system is remarkably similar. The most abundant lipids of myelin are cholesterol, the glycolipid galactosylceramide (GalC), and the phospholipid ethanolamine plasmalogen (EP), representing approximately 70% of total myelin lipids (Table 1, Norton and Poduslo, 1973; Chrast et al., 2011; Schmitt et al., 2015).

**Table 1:** Estimated lipid composition of the myelin membrane within the mammalian CNS.

**Lipid type**	**% in Myelin membrane**
Cholesterol	26%
Galactosylceramide (GalC)	23%
Sulfatide	4%
Ethanolamine Plasmogen (EP)	16%

*The table highlights the most common lipids present in myelin (Norton and Poduslo, 1973; Chrast et al., 2011; Schmitt et al., 2015)*.

### Cholesterol

Cholesterol is a critical component of the myelin sheath needed to preserve the complex arrangement of lipids and proteins of the myelin membrane. Along with GalC, cholesterol helps to form specialized domains along the membrane, the so-called “lipid rafts”, which contribute to protein trafficking and signaling, as well as myelin fluidity and stability (Decker, 2004; Saher et al., 2011; Montani, 2021). Because the blood-brain barrier prevents the uptake of circulating lipoprotein bound dietary cholesterol, cholesterol synthesis occurs *in situ* (Petrov et al., 2016). The rate of cholesterol synthesis in the CNS is at the highest during the first few weeks to months after birth, when OLs are engaged in active myelination. However, within the myelin membrane, there is long-term stability of cholesterol such that turnover occurs at the relatively slow pace of ~1 year in the young adult mouse brain and ~5 years in the human adult brain (Ando et al., 2003; Russell et al., 2009).

OL cholesterol synthesis is reliant on AcCoA generated from either glucose or ketone sources, although OLs show a preference for ketone bodies (Koper et al., 1981). AcCoA is formed from pyruvate (an end product of glycolysis) *via* pyruvate dehydrogenase or, in the case of ketones, the ketone body acetoacetate can be converted back into AcCoA *via* the enzyme β-ketoacyl-CoA. AcCoA then gets transformed into 3-hydroxyl-3-methylglutaryl-coenzyme A (HMG-CoA) and subsequently into mevalonate, which then goes on to produce lanosterol followed by a series of reactions that ultimately generates cholesterol. The intermediary reactions in this process are catalyzed by specific enzymes that require NADPH (a byproduct of the PPP) as a cofactor. Notably, the formation of mevalonate by HMG-CoA reductase is the irreversible, rate-limiting step for cholesterol biosynthesis (Petrov et al., 2016). HMG-CoA reductase is targeted by statin drugs to lower cholesterol production in the body. Given the widespread use of statins (e.g., simvastatin), several studies have used statins to assess the impact of cholesterol synthesis in OLs. Both OPCs and OLs that underwent prolonged treatment with simvastatin *in vitro* exhibited significant process retraction, increased cell death, and decreased mitochondrial activity whereas treatment with mevalonate or cholesterol itself was able to rescue the simvastatin-induced phenotype (Miron et al., 2007). Furthermore, severe hypomyelination has been observed in both white and gray matter tissue in mice engineered with OL-specific loss of *Fdft1*, the gene encoding squalene synthase, which catalyzes the first step of the cholesterol synthesis pathway. These *Fdft1* conditional knockouts displayed ataxia, tremors, and premature death (Saher et al., 2005). These and other studies have concluded that cholesterol is essential for myelin membrane growth and function, as well as for the survival of OPCs and OLs, which may be possibly mediated through signaling proteins contained within membrane cholesterol-enriched lipid rafts. In the absence of cholesterol, signaling proteins can become decoupled from lipid rafts, suppressing necessary signal transduction that mediates survival and other cellular processes in oligodendroglia (Bryant et al., 2009).

### Galactosylceramide

Galactosylceramide (GalC) is synthesized from a sphingolipid ceramide molecule. The UDP-galactose: ceramide galactosyltransferase (CGT) enzyme transfers UDP-galactose (UDP: uridine diphosphate) to ceramide, forming GalC. Interestingly, in CGT global knockout mice, no GalC production was detected and myelin instead contained high levels of *glucocerebroside*. Despite this compensatory mechanism, significant decreases in the number of myelinated axon segments in the spinal cord were observed. Unexpectedly, CGT deficient mice demonstrated a 50% increase in mature OLs, revealing a possible negative regulatory role for GalC in OL differentiation. A reduction in conduction velocity and insulative capacity of the myelin sheath was also noted, which led to progressive hindlimb paralysis, tremors, and even premature death (Coetzee et al., 1996; Bansal et al., 1999; Marcus et al., 2000). Problems with myelin compaction were also reported with the appearance of outward facing paranodal loops, outfolding of the myelin membrane, and incorrect myelin compaction (Bosio et al., 1998; Ishibashi et al., 2002).

The molecular structure of GalC has provided useful insights into its function. GalC and its sulfated form, sulfatide, are the most abundant glycolipids in the myelin membrane, accounting for 23% and 4% respectively, of the total myelin lipid mass (Norton and Cammer, 1984). GalC, like other galactosphingolipids, have longer, more saturated fatty acids than phospholipids and can participate in intermolecular hydrogen bonding, allowing them to form ordered membrane domains (Dicko et al., 2003). In fact, GalC is believed to interact with other sulfur enriched glycolipids such as sulfatide to form “glycosynapses” during the wrapping of the myelin sheath, contributing to the stability of myelin. These glycosynapses can interact with axonal ligands, extracellular matrix proteins, or even with each other across the apposed membranes to facilitate the transmission of transmembrane signals (Hakomori, 2002; Boggs et al., 2010). Liposomes containing GalC and sulfatide were added to OLs in culture to mimic facing pairs of extracellular surfaces of myelin or interactions between OLs processes. These liposomes were able to interact with glycosynapses to cause a disruption in the microtubule network and a concentrated redistribution of GalC on the extracellular side and MBP on the cytosolic side. However, as there is no clear role for the seGalC-MBP rich domains in OL signaling, future work is needed to understand their purpose (Boggs and Wang, 2001).

### Ethanolamine Plasmalogen

In the myelin membrane, the most abundant *phospholipid* belongs to the subclass plasmalogen. Plasmalogens are a type of glycerophospholipid that exhibit a vinyl-ether group on the first carbon of the glycerol molecule, with the predominant plasmalogen in OLs being ethanolamine plasmalogen (EP). EP synthesis begins in the peroxisome and is completed in the endoplasmic reticulum (Messias et al., 2018; Montani, 2021). Peroxisomal dihydroxyacetone phosphate acyltransferase (DHAPAT) enzyme is necessary for plasmalogen synthesis and loss of this enzyme results in ethanolamine deficiency, dysmyelination of the cerebellum, reduction in total MBP protein in both cortex and cerebellum, paranode disorganization, as well as a ~40% decrease in myelinated fibers of the EP deficient corpus callosum (Teigler et al., 2009). Future studies are still needed however to fully decipher the precise role EP has in OLs.

## Autophagy

One of the main ways that cells maintain metabolic homeostasis is through autophagy. During autophagy, cells form a double-membraned vesicle (the autophagosome) around components targeted for removal and reuse. Lysosomes containing degradative enzymes then fuse with the autophagosome to form the autolysosome. In this way, autophagy removes waste such as damaged organelles, proteins, and other cytoplasmic components for which the breakdown products become inputs to various metabolic processes (Eisenstein, 2014). Thus, autophagy is often thought of as the cell’s waste removal and recycling program. In terminally differentiated cells such as OLs, autophagy is critical in maintaining cellular health, as damaged components cannot be diluted out by cell replication. Additionally, in times of starvation or nutrient deprivation, autophagy can promote survival by breaking down cellular components to serve as metabolites for critical functions. Defects in autophagy have been connected to liver disease, aging, and neurodegeneration, and, while autophagy is generally considered to be beneficial, it can also contribute to tumorigenesis in nutrient-poor cancers. Thus, modalities aimed at upregulating autophagy should be approached with caution. Nevertheless, recent studies investigating the role autophagy plays in OL function have pointed to select benefits this “remove and reuse” mechanism can confer (Rabinowitz and White, 2010; Eisenstein, 2014).

To understand the role autophagy plays in OL function, Bankston and colleagues generated WT control and conditional knockout mice with the *Atg5* (Atg5 protein is necessary for the formation of the phagophore—the precursor to the autophagosome) deleted in mouse OPCs and OLs. Firstly, they found that oligodendroglia express autophagy markers at all stages but as OPCs transition to OLs they have substantial increasing levels of ATG5 and LC3B-II and corresponding decreases in p62, a pattern that is in accordance with autophagosome formation and lysosome fusion. Interestingly, the number of autophagic vesicles per area was significantly higher in OL processes than in OPCs, perhaps due to the rapid rate of growth OL processes experience during both differentiation and myelin membrane formation. To confirm this, immunostaining revealed that ATG5 and LC3B were present throughout the corpus callosum and in spinal cord white matter, as well as in myelinating co-cultures where ATG5 colocalized in myelin wrapped axon segments. Additionally, live cell imaging of tagged autophagosomes revealed the ability of OLs to engage in distal autophagosome formation as autophagosomes trafficked from the cell soma to its processes (Bankston et al., 2019).

The increased presence of autophagosomes in OLs and myelin sheaths suggested that autophagy activity may be necessary for myelination. Further experiments were conducted in mutant mice that had the *Atg5* gene selectively removed in OPCs. When compared to control, the *Atg5* mutant mice exhibited significantly fewer OPCs and OLs, with the few surviving OLs producing abnormal myelin that was uncompacted and with multiple myelin outfoldings. The *Atg5^−/−^*mice also displayed severe tremors by P12 followed by death by P15. Furthermore, a striking 75% reduction in MBP staining in the corpus callosum of the mutant mice was observed followed by a four-fold decrease in the percent of myelinated axons, strongly implicating autophagy as a key player in OL myelination. Similar findings were also observed when using pharmacological inhibitors of autophagy (KU55933 and Verteporfin) whereas, the use of Tat-beclin1, an autophagy *enhancer*, remarkably increased the number of myelin segments by more than two-fold when compared to vehicle controls or KU55933/Verteporfin treated OLs (Bankston et al., 2019). Autophagy has thus emerged as a critical player in OL function and myelinogenesis but additional study is clearly needed to fully understand this requirement.

## Signaling Events Regulating OL Metabolism

### Netrin-1 Signaling Promotes Mitochondria Elongation and Glycolysis

Understanding how both intrinsic and extrinsic signaling events can affect oligodendroglial metabolism will be key to the development of therapeutic agents to either enhance metabolic control of oligodendroglial functions or counter metabolic dysregulation. Several recent studies have shed light on some of the signaling pathways impacting OL metabolism. For example, netrin-1, a secreted protein that binds to the transmembrane receptor DCC (deleted in colorectal cancer), has recently been implicated in regulating OL mitochondrial dynamics and bioenergetics. Proteomic analysis and confocal imaging of netrin-coated beads in association with OLs revealed binding to paranodal enriched proteins DCC and neurofascin-1 (NF155). Similarly, binding to the dynein-1 heavy chain and kinesin light chain-1 (two major mitochondrial microtubule motor proteins) was observed, indicating OL netrin-1 may be associated with mitochondria trafficking. Indeed, super-resolution microscopy and transmission electron microscopy confirmed that netrin-1 was involved in mitochondria movement to distal regions of OL processes and paranodes. Furthermore, mitochondria elongation was enhanced by netrin-1 induced src family kinase (SFK) activation, and downstream inactivation of rho-associated protein kinase (ROCK). Mitochondrial elongation results from the fusion of multiple mitochondria organelles and as such can alter the metabolic dynamics of the cell. Live cell metabolic profiling showed that prolonged netrin-1 signaling (through SFK activation and ROCK inhibition) hyperpolarizes the mitochondrial inner membrane and increases glycolysis but not OXPHOS in primary OLs (Nakamura et al., 2021). Whether these netrin-1/SFK induced changes in OL metabolism can be beneficial in a demyelination paradigm with dysfunctional OLs remains to be determined but nevertheless, these findings present new pathways to pursue in the quest for next-generation oligodendroglia therapeutics.

### Oligodendrocyte NMDA Receptor Activation Increases Glucose and Lactate

OLs are known to provide trophic and metabolic support to the axons they ensheath, yet variations in synaptic strengths and neuronal firing rates can greatly alter the metabolic demand of axons. Thus, a key unanswered question is how OL adjust their ability to metabolically support axons in response to environmental cues (Mount and Monje, 2017; Bonetto et al., 2020). In a recent study, researchers were able to demonstrate that OLs “sense” neuronal activity through OL NMDA receptors, which can respond to the neurotransmitter glutamate (released upon neuronal firing) and then adjust the OLs ability to regulate axonal energy metabolism (Saab et al., 2016). For instance, stimulation of NMDA receptors with NMDA-acid and glycine (NMDA-A/Gly) in OL cultures triggered an increase in GLUT1 expression and trafficking to the membrane, leading to increased intracellular glucose and lactate concentrations as well as high lactate *release*. Selective deletion of the NR1 subunit [the glycine binding subunit critical to NMDA receptor function (Catts et al., 2015)] from OLs (NR1 cKO) conversely resulted in a strong reduction of GLUT1 transporters in OLs (e.g., an ~80% reduction in the optic nerve). GLUT1 deficiency in OLs negatively impacted the rate of myelin growth, which is metabolically controlled. Thinner and fewer myelinated axons were observed in the optic nerve of NR1 cKO mice by P18–P20. By adulthood (P70) myelination “caught up”, however, in the context of correct circuitry formation, delays in myelination may have detrimental effects on learning and memory in the postnatal brain by leading to aberrant synapse formation. Additionally, adult NR1 cKO mice (P50–P70) that were subjected to metabolic stress in the form of oxygen-glucose deprivation, had reduced conduction velocity (CV) in the optic nerve by as much as 50% compared to WT control, further demonstrating the role OL metabolism plays in regulating both OL dynamics and axon function. Remarkably, treatment with lactate for reperfusion of the nerve promoted CV similar to control levels, demonstrating axons readily use lactate to generate ATP which in itself can regulate axon CV (Massicotte et al., 2001; Saab et al., 2016).

### mTORC1 Signaling Regulates OL Myelination and Lipid Metabolism

All mammalian cells require sufficient nutrients for proper growth and maintenance. A major player in sensing and regulating cellular growth based upon nutritional availability is the mechanistic target of the rapamycin (mTOR) pathway. mTOR consists of two complexes, mTORC1, and mTORC2, both of which are capable of integrating nutrients such as glucose, oxygen, amino acids, and insulin to influence growth and proliferation (Sabatini, 2017). mTORC1 is functionally dependent on the regulatory associated protein of the mTOR (raptor) subunit whereas, mTORC2 activity is modulated by the rapamycin insensitive companion of the mTOR (rictor) subunit (Lebrun-Julien et al., 2014). The mTOR complex interacts with Akt kinase both in upstream and downstream signaling. Interestingly, overexpression of constitutively active Akt in mouse OLs resulted in significant CNS hypermyelination and as expected, elevated mTOR activity. However, when mTOR signaling was inhibited *via* chronic administration of rapamycin, the observed hypermyelination was significantly reduced in young adult mice (Narayanan et al., 2009). Recent findings have been able to further unravel the contributions each mTOR complex plays in CNS myelination with an emphasis being placed on mTORC1 for its part in modulating OL dynamics and myelin lipids (Lebrun-Julien et al., 2014).

Conditional knock-out mice lacking raptor, rictor, or a combination of raptor and rictor (raptor/rictor) in adult OLs were generated to study the effects of mTORC1 (raptor-dependent) and mTORC2 (rictor-dependent) in CNS myelin. *Both* raptor and rictor OL-knockouts resulted in reduced myelination when compared to control but the raptor knockout mice were persistently hypomyelinated into late adulthood whereas, rictor knockout mice underwent transient hypomyelination and restored their spinal cord myelin by p60, partly due to the compensation of raptor-driven mTORC1 activity. In double raptor/rictor knockouts, hypomyelination remained throughout adulthood (6 months) indicating mTORC1 is indeed critical for myelination. However, when analyzing OL dynamics in the raptor knockouts, the number of Olig2+, CC1+Olig2+, and PDGFRa+Olig2+ cells did not differ significantly from control as they did in the rictor knockouts. Interestingly, the double knockout raptor/rictor group did exhibit reductions in OPCs, mature OLs, and late progenitor to pre-myelinating OLs (characterized by Tcf-4+/Olig2+), revealing that the total mTOR complex (mTORC1 and mTORC2) contributes to oligodendroglia maturation. When analyzing levels of known transcription factors involved in OL differentiation (*Sox10* and *Myrf*), both the raptor/rictor and raptor mutant mice displayed strong decreases in transcript levels. In addition, the phosphorylation of 4E-BP1 and S6 kinase (downstream mTOR targets) was drastically reduced in the raptor and raptor/rictor mutants indicating mTORC1 activity is capable of regulating OL dynamics more than mTORC2 (Lebrun-Julien et al., 2014).

Because mTOR has a major role in lipogenesis, it was postulated that mTORC1 could also be in part responsible for controlling proper lipid biosynthesis in adult myelinating OLs (Soliman, 2011; Lamming and Sabatini, 2013). Similar to the hypomyelination phenotype observed during development, tamoxifen-inducible loss of raptor and raptor/rictor mutants in adult OLs displayed a significant reduction in myelin even 12 months post induction. Axon diameter was also reduced in these mutant mice. Although no changes were seen in sterol regulatory element-binding protein-1 (SREB1), transcription factors that regulate gene expression involved in lipid synthesis, the enzymatic activity of their main targets fatty acid synthase (FAS) and stearoyl-CoA desaturase-1 (SCD1), decreased in raptor and raptor/rictor mutants (Lebrun-Julien et al., 2014; Shimano and Sato, 2017). Interestingly, alterations in lipid composition were observed in the raptor mutant group with total levels of cholesterol, ceramide, sphingomyelin, and glycosylceramide (critical component of myelin—see Section “Reactive Oxygen Species”) severely reduced in the whole spinal cord (Lebrun-Julien et al., 2014). Taken together, these results indicate that mTORC1 activity of the mTOR complex is predominantly responsible for affecting OL lipid biosynthesis as well as myelin growth and maintenance.

## Exogenous Modulators of OL Metabolism: From Drugs to Dietary Therapies

### Donepezil Promotes OL Differentiation and Increases Brain Glucose Metabolism

The ability to exogenously regulate oligodendroglial metabolism has implications for a host of neurodegenerative diseases. For instance, in sporadic AD, a strong correlation exists between impaired brain glucose metabolism and disease onset and progression (Saito et al., 2021). More specifically, when analyzing gene expression data in post-mortem AD brains *via* RNA sequencing, impairments in both glycolytic and ketolytic genes were observed, more so in OLs than in neurons, astrocytes, or microglia, suggesting that regional OL hypometabolism may contribute much more to disease pathology than originally believed (Saito et al., 2021). In support of a more central role for OL dysfunction in AD, there is evidence that the demyelination precedes the toxic beta-amyloid and tau pathologies that lead to synaptic dysfunction and symptoms of cognitive decline in AD (Papuć and Rejdak, 2018). One drug approved for mild to moderate AD that has been shown to positively influence AD outcomes is donepezil. A potent and selective inhibitor of acetylcholinesterase (AchE), donepezil helps to reduce the breakdown of acetylcholine in the body thereby, prolonging the activity of cholinergic neurons in the brain and thus slowing down the neuronal loss normally observed in AD (Chen and Mobley, 2019). Recent research has revealed that donepezil might also exert its anti-AD therapeutic effects *via* its impact on OLs. Cui and colleagues conducted experiments on neural progenitor cell (NPC)-derived OPCs treated with varying concentrations of donepezil and found that donepezil promoted OPC-to-OL differentiation in a dose-dependent manner with higher concentrations nearly doubling the number of differentiated OLs relative to those in control cultures. Furthermore, this effect was limited to donepezil as other AchE inhibitors used in AD therapy such as rivastigmine, tacrine, and huperzine A did not result in any significant changes in the number of OLs. When donepezil was administered to mice undergoing active remyelination following cuprizone-mediated demyelination, by 2 weeks the donepezil treated group had a significant increase in the number of myelinated axons per field as well as thicker myelin, as defined by a significant decrease in the *g ratio* (the inner axonal diameter/outer axonal diameter; Cui et al., 2019). Interestingly, in a separate randomized, double-blinded, placebo-controlled study assessing the effect of donepezil in 28 patients with mild to moderate AD over a 24-week period, overall brain glucose metabolism was maintained within 0.5% of baseline levels in the treatment group whereas, the placebo group experienced an average 10.4% decline in brain glucose metabolism. In the parietal, temporal, and frontal lobes (areas where white matter lesions are often observed in AD patients), a slight but significant increase in glucose metabolism was also observed in AD patients on donepezil, which may contribute to the therapeutic effect of the drug (Tune et al., 2003). Whether or not donepezil’s pro-myelination effects are mediated through metabolic changes in OLs remains to be seen but the data gathered thus far presents a hypothesis worth investigating.

### The Antipsychotic Drug Clozapine Increases Glycolytic Activity and Myelin Synthesis

OL dysfunction in SCZ has recently been linked to cerebral dysconnectivity, where white matter anomalies, decreased OL number, and the breakdown of neuronal circuitry across varying brain regions are observed (Schmitt et al., 2011; Steiner et al., 2014). Moreover, elevated glucose levels and reduced lactate were identified in the cerebrospinal fluid of first onset, drug-naive SCZ patients (Holmes et al., 2006). Additionally, decreased expression in glycolytic and glycogen synthesis enzymes was observed and genetic studies have identified linkages between genes involved in glucose metabolism with an elevated risk of SCZ (Prabakaran et al., 2004; Stone et al., 2004). Taken together, SCZ is a disease closely tied to both OL dysfunction and impaired glucose metabolism of the brain. Clozapine, an atypical antipsychotic drug used to mitigate symptoms in SCZ, has antagonistic effects on serotonin (5-HT) and dopamine receptors, yet new evidence suggests that clozapine’s therapeutic efficacy may in part stem from its ability to upregulate key metabolic mediators in OLs, possibly by disinhibiting decreased activation of the MAPK/ERK pathway following 5-HT stimulation (Meltzer, 1994; Masson et al., 2012; Steiner et al., 2014). In support of this idea, clozapine treatment of OLN-93 cells (an OL cell line) led to increased glycolytic activity and lactate production as well as increases in GalC and myelin lipid synthesis (Steiner et al., 2014). A notable and significant increase in intracellular glucose was observed in the clozapine treatment group but the number of GLUT1 transcripts was not changed. Similarly, lactate concentrations were increased in both intracellular homogenates and extracellular media, suggesting upregulation of glycolysis in treated cells. However, no significant changes in mitochondrial oxygen consumption were noted. Interestingly, clozapine treatment led to elevated acetyl-CoA-carboxylase (ACC) activity, a key rate-limiting enzyme that converts AcCoA into the fatty acid malonyl-CoA needed for downstream lipogenesis (Brownsey et al., 2006). Although pre-clinical data with clozapine looks promising for treating white matter pathologies, a recent phase 1, randomized, blinded, placebo controlled clinical trial in patients with progressive MS (pMS), concluded that clozapine was not well tolerated as patients experienced spastic ataxic gait and worsening mobility, which resolved after drug cessation (La Flamme et al., 2020). Thus, additional studies are needed to achieve a better understanding of this promising drug’s pharmacodynamics and other parameters in the context of human disease.

### Metformin-Induced AMP-Kinase Activation Influences OL Function

The anti-hyperglycemic drug metformin has been used extensively to treat Type-2 Diabetes Mellitus and is globally the most prescribed anti-diabetic drug. Metformin is believed to exert its therapeutic effect by indirectly altering the metabolic state of cells. Due to its polarity, metformin entry into the cell is facilitated by members of the organic cation transporter (OCT) family. Following entry, metformin binds to complex I in the mitochondria to inhibit its function, resulting in decreased ATP output. This causes a shift in the cellular ATP:AMP ratio, which is detected by the enzyme complex AMP-kinase (AMPK; Zhou et al., 2001). In addition to its role as the cells’ “metabolic sensor”, AMPK is a master regulator of many signaling pathways, including the mTOR pathway (Sabatini, 2017). When activated, AMPK will suppress many energy intensive anabolic processes that require ATP (e.g., gluconeogenesis and protein synthesis) and turn on catabolic processes such as beta-oxidation to liberate more cellular ATP. In the context of type II diabetes, metformin-induced activation of AMPK leads to increased trafficking of GLUT transporters to increase glucose uptake and glycolysis, thereby, lowering blood glucose (Pernicova and Korbonits, 2014). Additionally, AMPK activation also decreases cyclic AMP, which in turn through downstream signaling increases the activity of phosphofructose kinase-1, thus leading to elevated levels of glycolysis to generate ATP and downregulation of gluconeogenesis (Pernicova and Korbonits, 2014). However, it is important to note that metformin’s effect on cellular metabolism is only well understood in hepatocytes where a large concentration of OCT1 transporters is localized in the body. Nevertheless, active research is exploring the potential other uses for metformin in metabolopathies such as cancer. Given the presence of other OCT transporters in the brain, mainly OCT3 (believed to be a functional but less potent transporter of metformin; Shirasaka et al., 2016; Koepsell, 2021), several groups have begun to investigate metformin’s effect on the CNS. New findings regarding the relationship between metformin and myelin are discussed below (Rotermund et al., 2018).

#### Metformin Reduces Degeneration of RGC Neurons and Increases Myelin Thickness

Neurodegenerative diseases such as PD, AD, and glaucoma can lead to the loss of retinal ganglion cells (RGC). Photoreceptor cells convey visual information to RGC neurons whose myelinated axons form the optic nerve responsible for engaging visual cortex processing (Etxeberria et al., 2016; Kim et al., 2021). By using the DNA alkylating agent N-ethyl-*N-*nitrosourea (ENU) to induce retinal photoreceptor degeneration in rats, Eltony et al. (2021) assessed whether metformin was neuroprotective. Daily ENU treated retina developed pronounced photoreceptor degeneration with vacuolations and loss of nuclei, followed by RGC death and a reduction in myelinated axons. Interestingly, when undergoing daily metformin concurrently (metformin given intraperitoneally @ 200 mg/kg), the severity of photoreceptor degeneration was reduced. Additionally, the diameter of the optic nerve fiber and the thickness of its myelin sheath increased significantly by >two-fold when compared to the ENU+ only group, although the metformin+ENU group did not match the levels seen in the untreated controls. Metformin also led to significant reductions in inducible nitric oxide synthase (iNOS), a proinflammatory agent induced by microglia, and the apoptotic marker caspase-3, whose activity is modulated by AMPK (Villanueva-Paz et al., 2016; Eltony et al., 2021). However experiments to measure OL dynamics or its metabolism were not conducted, thus leaving open the question of whether changes in myelin were due to alterations in OL metabolism and possibly myelin repair (Eltony et al., 2021).

#### Metformin Increases Neurotrophic Factors and Stimulates Remyelination *via* AMPK

Neurotrophic factors (NTFs) are small molecules that help to promote and regulate the survival, growth, and plasticity of cells in the nervous system (Johnson and Tuszynski, 2008). In addition to their known effects on neurons, NTFs such as brain-derived neurotrophic factor (BDNF), ciliary neurotrophic factor (CNTF), and nerve growth factor (NGF) also act upon OLs where they can promote survival and differentiation (Pöyhönen et al., 2019). However, the key effectors involved in OL NTF signaling and the degree to which NTFs can influence repair in diseased OLs remain poorly understood. Using cuprizone (a copper chelating agent that inhibits complex IV of the ETC) to induce demyelination, Houshmand et al. (2019) reported that treatment with metformin-induced upregulation of BDNF, CNTF, and NGF, and downregulated neurite outgrowth inhibitor (NogoA) mRNA in OLs, which led to an increase in OPC proliferation as well as OL differentiation. Interestingly, these pro-OL effects were absent in the combined cuprizone (−) and metformin (+) group, suggesting that the increase observed in these neurotrophic factors may only be linked to periods of OL metabolic dysfunction (Faizi et al., 2016; Houshmand et al., 2019). Neurodegenerative diseases such as PD, HD, and MS all exhibit decreases in the trophic factor BDNF which in part contributes to neuronal loss (Sohrabji and Lewis, 2006; Scalzo et al., 2010; Bathina and Das, 2015). Yet BDNF has also been found to promote OL myelination through its effects on Fyn kinase (a member of the Src family of kinases) signaling, which itself has been proposed to regulate lipid metabolism *via* its upstream effects on AMPK (Vatish et al., 2009; Peckham et al., 2016). Thus, the metformin-to-BDNF axis may have a dual effect in the context of myelin loss, both promoting OL intrinsic repair mechanisms as well as providing needed trophic support to axons.

#### Metformin Rejuvenates Aged OPCs to Increase CNS Remyelination

Aging is associated with a metabolic decline in the brain. In both human and animal studies, aging has been linked to reduced expression of glucose transporters and altered levels of glycolytic and OXPHOS enzymes in the brain (Camandola and Mattson, 2017). Decreases in ATP levels are also observed in aging white matter and are accompanied by ultrastructural changes in mitochondria (Stahon et al., 2016). In the context of neurodegenerative diseases such as MS, aging contributes to impairments in the endogenous remyelination capacity that wanes during disease progression. For instance, remyelination relies upon the recruitment and differentiation of OPCs at the lesion site, both of which decline with age. A recent study by Neumann et al. (2019) found that, when compared to OPCs from young adult rats (2 to 3 months old), OPCs isolated from adult rats aged 20 to 24 months were largely unable to differentiate into OLs when stimulated with triiodothyronine (T3), a well-known OL differentiation. Thus, employing strategies that can counter the effects of aging may be able to provide aged OPCs with a more “youthful” phenotype that can overcome differentiation deficits. Neumann et al. (2019) focused on alternate day fasting (discussed below) and administration of metformin. In addition to being considered a fasting mimetic (fasting also activates AMPK), metformin is also thought of as a geroprotective agent. A systematic review and meta-analysis study conducted by Campbell et al. (2017) revealed that the use of metformin increased health and lifespan by reducing both all-cause mortality and diseases of aging through either its effect on ROS, mTOR, and AMPK signaling or lowering inflammation and DNA damage. In fact, when Neumann and colleagues treated aged OPCs with 100 μM metformin, they found the aged OPCs reverted to a “younger” state and significantly increased their responsiveness to differentiation factors. They attributed these findings to significant reductions in DNA damage and the senescent marker *Cdkn2a*, as well as demonstrated that metformin’s effects required AMPK signaling since inhibition of AMPK using dorsomorphin or knockout of *Prkaa2* (the gene encoding for the catalytic AMPK α2 subunit) ameliorated the pro-OL effects with metformin treatment (Neumann et al., 2019).

### Dietary Changes Influence OL Function

Systemic metabolic changes can also be achieved through alterations in dietary intake. For instance, a diet high in glucose, carbohydrate, and fat can lead to impaired glucose metabolism, dyslipidemia, and hyperinsulinemia, which collectively can cause a metabolic syndrome with symptoms such as hypertension, fibrosis, and inflammation in the heart, liver, and kidneys (Panchal et al., 2011). Brain development is also often affected by dietary changes. In fact, when subjecting piglets to sustained iron deficiency for the first month of life, white matter development was significantly impaired throughout with the greatest reduction seen in the corpus callosum as evident by changes in its fractional anisotropy and anatomical width (Leyshon et al., 2016). In contrast, iron homeostasis is critical for proper CNS function, particularly for OPCs and mature OLs, which have the highest cellular iron in the brain, utilizing iron as a cofactor for enzymes involved in glucose oxidation and lipid and cholesterol synthesis that make up the myelin sheath (e.g., lipid saturases and lipid dehydrogenases (Stephenson et al., 2014; Cheli et al., 2020).

Dietary fasting is also capable of inducing changes in oligodendroglia. In the previously mentioned study (see “Metformin Rejuvenates Aged OPCs to Increase CNS Remyelination” Section), Neumann and colleagues investigated the potential alternate day fasting (ADF) may have on OPC dynamics. As noted previously, aged OPCs were unable to undergo efficient differentiation as well as have decreased ATP production and basal respiration rates. ADF is capable of altering the aging processes by restricting caloric intake both of which induce changes to the body’s metabolism (Heilbronn and Panda, 2019). The ADF group was given access to food on alternate days for 6 months compared to the control group which was able to feed *ad libitum*. Upon cessation of the ADF fasting period, demyelination was induced in cerebellar white matter by means of focal ethidium bromide injection. Immunohistological analysis revealed a two-fold increase in the number of mature myelinating cells (identified with the markers CC1 and MBP) in the ADF group when compared to the “old” controls. This finding supports the idea that ADF may prove to be beneficial for WM deficiencies, but more studies are warranted to fully assess its therapeutic efficacy in humans.

Other dietary therapies such as the ketogenic diet (KD) have been recognized for their positive effects on disease. The KD, first described in 1921, is well known for its antiseizure effects and as such is used to treat intractable epilepsy in both adults and children (Vining, 1999). The traditional KD consists of high fat, low carbohydrate, and limited but adequate protein to allow the body to transition away from using glucose for “fuel” and instead to using ketones from fat. While the exact underlying cellular and molecular mechanisms of the KD remain elusive, it is being explored for treatment in AD, PD, and amyotrophic lateral sclerosis (ALS) because of its positive effects on the brain. Accruing evidence, however, suggests that the KD may mechanistically be working through activation of ATP sensitive potassium channels in cells of the CNS and/or by inhibition of the mTOR pathway which interestingly is linked to myelination and AMPK activity (see Section “mTORC1 Signaling Regulates OL Myelination and Lipid Metabolism”). Therefore, it seems plausible that the KD should be able to alter OL dynamics as well, a concept Stumpf and others set out to investigate using a mouse model of Pelizaeus-Merzbacher disease (PMD), a hereditary leukodystrophy. PMD most often occurs *via* a duplication in the X-linked myelin gene, proteolipid protein 1 (*PLP1*), causing PLP-cholesterol accumulation and subsequently impaired protein-lipid transport to the myelin sheath. As a result, PMD presents with pathological hypomyelination and symptoms of nystagmus, spasticity, ataxia, and impaired cognitive development. Furthermore, late stage PMD is accompanied by progressive loss of OLs, axonal degeneration, and cortical atrophy, some of which were reversed after starting a KD (Stumpf et al., 2019).

PMD and control mice were fed either a high fat/low carbohydrate KD or a standard control diet for 2–12 weeks while monitoring ketone bodies such as beta-hydroxybutyrate, which in the brain helps facilitate cholesterol synthesis and is essential for myelin membrane growth (Koper et al., 1981; Saher et al., 2005). As expected, the mice on the KD saw a five-fold increase in blood ketone levels and a 70% drop in serum glucose in as little as 7 days from KD onset when compared to control mice. Remarkably, PMD mice on the KD significantly increased *PLP1* mRNA expression followed by increases in the number of Olig2+ cells and mature OLs. Furthermore, these changes in OL dynamics also resulted in more myelinated axons in the PMD-KD mice with the *g-ratio* matching that seen in WT control mice. Most importantly, these positive changes in myelination were able to restore normal function in the PMD mice. When assessing motor performance with the elevated beam test and rotarod test, control diet PMD mice exhibited progressive worsening of motor functions over time whereas, the PMD-KD mice retained motor fitness at WT levels. Independent of PMD pathology, the researchers confirmed the pro-OL/pro-myelin effects of the KD diet were also applicable in the cuprizone model of adult remyelination where during the cuprizone off/remyelination phase, mice on a KD diet saw accelerated repair 2 weeks into remyelination, evident by significant increases in mature OLs and reduction in the number of slips on the beam motor test (Stumpf et al., 2019). Hence when taken together, entering into a state of ketosis may prove beneficial for patients coping with WM diseases and in fact several clinical trials are currently underway to determine if the KD diet can reverse and repair WM damage in the CNS (ClinicalTrials.gov Identifier: NCT03718247, NCT04820478, NCT03509571, CT04530032, and NCT04701957).

## Conclusion

**Figure 2:**
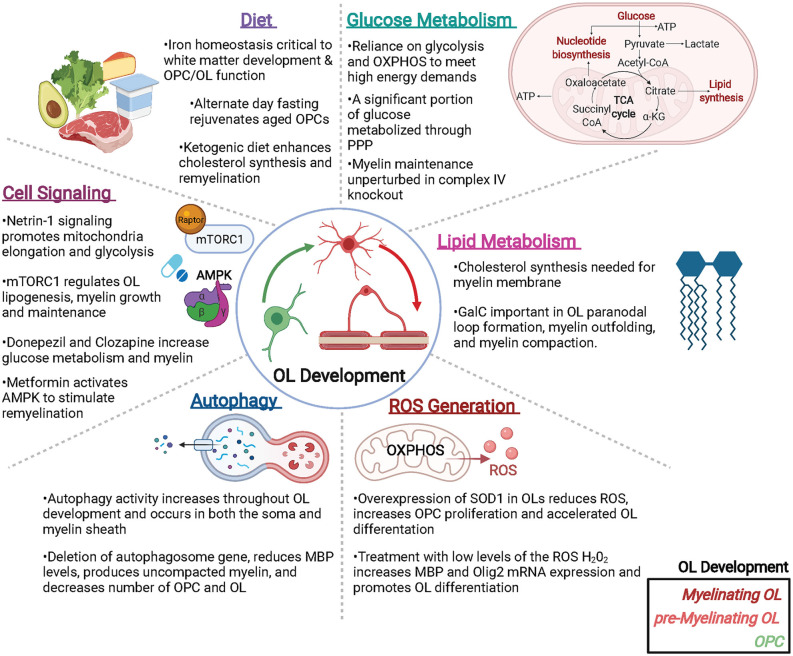
Metabolic factors impacting oligodendroglial development and function. Oligodendrocytes (OLs) are metabolically active cells that both myelinate and provide trophic support to neurons, as well as supply metabolites to neurons and other cells of the CNS. Many metabolic factors can alter OL function including (but not limited to): glucose and lipid metabolism, reactive oxygen species (ROS) that can generate pro- or anti-OL effects, autophagy, cell signaling events, and diet. Abbreviations: PPP, pentose phosphate pathway; OXPHOS, oxidative phosphorylation; SOD, superoxide dismutase; mTORC1, mammalian target of Raptor complex I. Created with BioRender.com.

Oligodendroglia are specialized cells whose metabolism is uniquely tuned to support its functions. From developmental myelination to adaptive myelination to myelin repair, OLs regulate a host of metabolic components to meet the energetic demands of both themselves and the axons they support. Disruptions in OL metabolism are likely to contribute to many neurological diseases including SCZ, PD, AD, and MS, all of which may benefit from next generation therapeutics that target or override OL metabolic pathways. Here we have summarized some of the key recent studies that address metabolic elements important to oligodendroglial function, such as the rate of glycolysis, lipid metabolism, ROS generation, and autophagy, as well as the intrinsic and extrinsic signaling pathways that regulate OL metabolism (Figure 2). While much has been revealed by these recent exciting advances, a great deal of future research is needed to fill in the gaps in knowledge between OL metabolism and OL function, both in health and in disease.

## Author Contributions

MN and HC wrote and edited the manuscript. All authors contributed to the article and approved the submitted version.

## Funding

This work was supported by funding from National Institutes of Health (HC; NS114769), the National Multiple Sclerosis Society (HC; RG-1607-25279), and a Turner Dissertation Fellowship (MN).

## Conflict of Interest

The authors declare that the research was conducted in the absence of any commercial or financial relationships that could be construed as a potential conflict of interest.

## Publisher’s Note

All claims expressed in this article are solely those of the authors and do not necessarily represent those of their affiliated organizations, or those of the publisher, the editors and the reviewers. Any product that may be evaluated in this article, or claim that may be made by its manufacturer, is not guaranteed or endorsed by the publisher.
